# Human bone marrow mesenchymal stem cells-derived exosomes alleviate liver fibrosis through the Wnt/β-catenin pathway

**DOI:** 10.1186/s13287-019-1204-2

**Published:** 2019-03-18

**Authors:** Xiaoli Rong, Junzhi Liu, Xia Yao, Tiechao Jiang, Yimin Wang, Feng Xie

**Affiliations:** 1 Department of Clinical Laboratory, China-Japan Union Hospital of Jilin University, 126 Xiantai St., Changchun, 130033 Jilin China; 2 The Scientific Research Center, China-Japan Union Hospital of Jilin University, 126 Xiantai St., Changchun, 130033 Jilin China; 3 Department of Quality Control, China-Japan Union Hospital of Jilin University, 126 Xiantai St., Changchun, 130033 Jilin China; 4 Department of Anesthesiology, Affiliated Hospital of Changchun University of Traditional Chinese Medicine, 1478 Gongnong Road, Changchun, 130117 Jilin China; 5 Department of Cardiology, China-Japan Union Hospital of Jilin University, 126 Xiantai St., Changchun, 130033 Jilin China

**Keywords:** hBM-MSCs, Exosomes, Liver fibrosis, Wnt/β-catenin

## Abstract

**Background:**

Mesenchymal stem cells (MSCs) are increasingly being applied as a therapy for liver fibrosis. Exosomes possess similar functions to their parent cells; however, they are safe and effective cell-free reagents with controllable and predictable outcomes. In this study, we investigated the therapeutic potential and underlying molecular mechanism for human bone mesenchymal stem cells-derived exosomes **(**hBM-MSCs-Ex) in the treatment of liver fibrosis.

**Methods:**

We established an 8-week CCl_4_-induced rat liver fibrosis model, after which, we administered hBM-MSCs-Ex in vivo for 4 weeks. The resulting histopathology, liver function, and inflammatory cytokines were analyzed. In addition, we investigated the anti-fibrotic mechanism of hBM-MSCs-Ex in hepatic stellate cells (HSCs) and liver fibrosis tissue, by western blotting for the expression of Wnt/β-catenin signaling pathway-related genes.

**Results:**

In vivo administration of hBM-MSCs-Ex effectively alleviated liver fibrosis, including a reduction in collagen accumulation, enhanced liver functionality, inhibition of inflammation, and increased hepatocyte regeneration. Moreover, based on measurement of the collagen area, Ishak fibrosis score, MDA levels, IL-1, and IL-6, the therapeutic effect of hBM-MSCs-Ex against liver fibrosis was significantly greater than that of hBM-MSCs. In addition, we found that hBM-MSCs-Ex inhibited the expression of Wnt/β-catenin pathway components (PPARγ, Wnt3a, Wnt10b, β-catenin, WISP1, Cyclin D1), α-SMA, and Collagen I, in both HSCs and liver fibrosis tissue.

**Conclusions:**

These results suggest that hBM-MSCs-Ex treatment could ameliorate CCl_4_-induced liver fibrosis via inhibition of HSC activation through the Wnt/β-catenin pathway.

**Electronic supplementary material:**

The online version of this article (10.1186/s13287-019-1204-2) contains supplementary material, which is available to authorized users.

## Background

Liver fibrosis is characterized by the excessive accumulation of extracellular matrix (ECM) [1]. Such chronic liver damage is a serious health problem worldwide [2]. However, there is currently no effective therapy for liver fibrosis, except for removal of the underlying etiology or liver transplantation [3]. Recently, a number of studies have evaluated the ability of mesenchymal stem cells (MSCs) to reduce liver fibrosis and improve liver function [4–6]. Different sources of MSCs have highlighted the anti-fibrotic potential of this cell type in animal models [7–9]. In addition, clinical trials have demonstrated that MSC transplantation is effective in the treatment of human liver fibrosis [10]. However, the risk for iatrogenic tumor formation and cellular rejection in MSC transplantation remains unresolved [11]. Recent studies have suggested that a novel cell-free therapy, MSCs-secreted exosomes, may present a new therapeutic strategy due to their advantages over MSCs [12–14].

Exosomes (30–100 nm) are small membrane-bound vesicles derived from multivesicular bodies [15]. They are less complex and smaller in size than their parent cells and thus are easier to produce and store [16]. Moreover, they present no risk for tumor formation and are less immunogenic [17]. Multiple recent studies have demonstrated that exosomes derived from MSCs are beneficial in the treatment of hepatic diseases [18, 19]. A previous study found that gene-modified human bone marrow-derived mesenchymal stem cells (hBM-MSCs) could attenuate liver fibrosis in rats by downregulating the Wnt signaling pathway [20]. Additional studies indicate that hBM-MSCs-Ex have therapeutic promise in cardiovascular, bone, and blood diseases [21–23]. However, the application of hBM-MSCs-Ex in the treatment of liver fibrosis has not been reported, and the potential therapeutic mechanism remains unclear.

The aim of the present study was to investigate whether transplantation of hBM-MSCs-Ex reduces liver fibrosis in a CCl_4_-induced liver fibrosis model in rat. We further analyzed the involvement of Wnt/β-catenin signaling on hBM-MSCs-Ex-induced anti-fibrosis in vitro. Our results provide the first evidence that hBM-MSCs-Ex effectively alleviate liver fibrosis through the Wnt/β-catenin pathway. We believed that hBM-MSCs-Ex could provide a new avenue for the therapeutic treatment of hepatic fibrosis disease.

## Methods

### Cell culture

The hBM-MSCs were purchased from the Chinese Academy of Medical Sciences, China. Cells were cultured in DMEM (Gibco, Grand island, USA) supplemented with 10% FBS (Gibco, Australia), 500 U/ml penicillin, and 500 μg/ml streptomycin (Invitrogen, Shanghai, China) at 37 °C, with saturated humidity and 5% CO_2_. Morphological observation of hBM-MSCs was performed by phase contrast microscopy (Eclipse TE200; Nikon, Tokyo, Japan). Cells at the fifth passage were used for this study [24]. Hepatic stellate cells (HSCs) were acquired from Dr. Zhang in our lab. The same culture conditions were used for HSCs as for hBM-MSCs. Cells were passaged using trypsin (Sigma, San Francisco, USA) and stored in liquid nitrogen in freezing medium (DMEM: FBS: DMSO = 6:3:1).

### Exosome purification and characterization

The purification of hBM-MSCs-Ex involves several centrifugation and ultracentrifugation (Himac cp80wx/P70A-980) steps, as described previously [25]. Briefly, hBM-MSCs were cultured in serum free medium (SFM, Gibco, Grand island, USA) for 2 days. The conditioned medium was first filtered using a 0.1-μm filtering device, followed by VACUCAP filtering for conical tubes (Pall Laboratory/VWR, USA). The supernatant was concentrated with a 100-kDa molecular weight cutoff (MWCO) hollow fiber membrane (Millipore, Billerica, MA, USA), at 1000 g, 30 min. The concentrated supernatant was loaded onto a 30% sucrose/D2O cushion (5 ml, density 1.210 g/cm^3^), and ultra-centrifuged at 100,000*g*, 3 h. The exosome-enriched fraction was collected, washed with PBS three times, and centrifuged at 1500*g*, 30 min with 100-KDa MWCO. Purified exosomes were passed through a 0.22-μm filter and stored at − 80 °C. The protein concentration of the concentrated exosomes was determined using a bicinchoninic acid (BCA) protein assay kit (Beyotime, Shanghai, China). The presence of the exosomal markers CD9, CD63, CD81, TSG101, and Alix (Abcam, Cambridge, UK) were determined using western blotting. Purified exosomes were confirmed by transmission electron microscopy.

### Transmission electron microscopy

The hBM-MSCs-Ex obtained after differential centrifugation of conditioned cell-culture medium was suspended in PBS. Ten micrograms of exosome suspension was loaded onto formvar carbon-coated 200 mesh copper grids for 10 min at room temperature. Excessive fluid was drained with filter paper. Adsorbed exosomes were negatively stained with 1% phosphotungstic acid for 5 min. Then, the air-dried exosome-containing grids were observed by transmission electron microscope (JEM-1400PLUS, Japan) operating at 100 kV.

### Nanoparticle tracking analysis (NTA)

The NanoSight NS300 (Cambridge, MA, USA) was used to determine the concentration and size distribution of purified airway hBM-MSCs-Ex. Polystyrene latex microspheres (Malvern Instruments Ltd., Malvern, UK) of 100 nm diameter were used to calibrate the instrument. Then, hBM-MSCs-Ex were diluted with PBS 1:1000 to make a final volume of 1 ml and loaded into a 1-ml syringe. The syringe was placed on a syringe pump, and hBM-MSCs-Ex were infused at a flow rate of 25 μl/s at room temperature. The camera level was set to 7, gain to 1, and detection threshold to 5. A total of five videos were acquired with a duration of 1 min per video. A minimum of 2000–4000 exosomes/video were processed.

### CCl_4_-induced liver fibrosis in rats

Liver fibrosis was induced in Sprague Dawley (SD) rats (8-week old, female, 200 g). All protocols and procedures were approved by the Animal Experiment Ethic Committee of Jilin University (Approval NO. 201802084). All experimental procedures were in accordance with the Chinese legislation regarding experimental animals. Rats were administered with an intraperitoneal injection of CCl_4_ at a dose of 30% CCl_4_ 3 ml/kg body weight twice weekly in olive oil. Eight weeks later, CCl_4_ treated rats were randomly assigned into four groups (*n* = 12): the liver fibrosis group rats were injected with PBS alone; the hBM-MSCs group (positive control) rats were injected with 1 × 10^6^/500 μl hBM-MSCs through the tail vein; the hBM-MSCs-Ex group rats were injected with 250 mg hBM-MSCs-Ex (harvested from 1 × 10^6^ hBM-MSCs) in 500 μL PBS through the tail vein; the sham group rats were treated with PBS alone. After 4 weeks, serum was taken to assay liver function and the livers were collected. Livers were divided into three parts for immediate protein and RNA isolation, preservation in 10% formalin for histological examination, and freezing at − 80 °C for hydroxyproline (Hyp) and malonaldehyde (MDA) assay.

### Histopathological analysis

Liver tissues were processed for paraffin embedding by slicing into 4-μm sections. The sections were stained with hematoxylin and eosin (H&E), Masson, and Sirius red according to standard protocols. To analyze the extent of liver fibrosis, randomly selected fields of Masson sections were captured from each animal. The collagen stained area was calculated via Image-Pro Plus. The degree of hepatic fibrosis was assessed using the Ishak modified scoring system. The Ishak scoring criteria ranges from 0 to 6 (0 = no fibrosis, 6 = cirrhosis): mild fibrosis (Ishak, 0–2) to severe fibrosis (Ishak, 3–6) [26]. The liver sections were analyzed in ten random fields per section and twelve sections in total (*n* = 12 rats) for quantification of immunohistochemistry (IHC) results. The IHC results were calculated via Image-Pro Plus.

### Biochemical analysis

The serum levels of alanine aminotransferase (ALT), aspartate aminotransferase (AST), total protein (TP), total bilirubin (TBIL), alkaline phosphatase (ALP), and gamma glutamyl transpeptidase (γ-GT) were assessed using the Automated Biochemical Analyzer (AU-680, Beckman, Germany). Liver homogenate (10%, *w*/*v*) was prepared by homogenizing the right lobe of liver on ice in 150 mM Tris-HCl buffered saline (pH 7.2; Sigma-Aldrich) using a polytron homogenizer (PT3100D; Kinematical, Lucerne, Switzerland). The levels of hydroxyproline (Hyp) and malondialdehyde (MDA) in liver tissue were measured using kits (NanJing JianCheng Bioengineering Institute, A030-2, A003-1, Nanjing, China) according to the manufacturer’s instructions.

### Quantitative real-time PCR (qRT-PCR)

Total RNA was isolated from liver tissue using Trizol reagent (Invitrogen, Shanghai, China) according to the manufacturer’s protocol. Then, 1 μg total RNA was reverse-transcribed to give cDNA, which was used as the template, and combined with standard SYBR premix Ex Taq (Invitrogen, Shanghai, China) on the Real-Time PCR Detection System (Roche, Basel, Switzerland). The following primers were used: GAPDH forward, 5′-agacagccgcatcttcttgt-3′, reverse, 5′-cttgccgtgggtagagtcat − 3′; IL-1 forward, 5′-atttccgccttccagagaat-3′, reverse, 5′-gagtctcatgggggaattga; IL-2 forward, 5′-aaactccccatgatgctcac-3′, reverse, 5′-gaaatttccagcgtcttcca-3′; IL-6 forward, 5′-ccggagaggagacttcacag-3′, reverse, 5′-acagtgcatcatcgctgttc-3′; IL-8 forward, 5′-atgagacactgtggctgtgc-3′, reverse, 5′- actgctggagaccaggaaga-3′; IL-10 forward, 5′-cctgctcttactggctggag-3′, reverse, 5′-tgtccagctggtccttcttt-3′; TNF-α forward, 5′-gtgacgtggagttgggtctt-3′, reverse, 5′-gagtccgtcttggtcagagc-3′. GAPDH served as the internal control, and experiments were conducted in triplicate. All reactions were performed in triplicate and the data were analyzed using the 2^-ΔΔCt^ method.

### Immunohistochemistry analysis

Immunohistochemistry (IHC) was performed in accordance with the manufacturer’s instructions (Cat. no. kit-9710; Fuzhou Maixin Biotech Co., Ltd., Fuzhou, China). Briefly, the liver sections were deparaffinized and then rehydrated in a descending alcohol series. Antigen retrieval was performed by heating the sections for 30 min at 95 °C in 1 mM EDTA buffer (pH 8.0). Thereafter, sections were added to 3% H_2_O_2_ for 15 min and blocked with 10% normal goat serum (Cat. no. kit-9710; Fuzhou Maixin Biotech Co., Ltd., Fuzhou, China) at 37 °C for 1 h. Sections were then incubated with primary antibodies (1:500 dilution) against hepatocyte nuclear factor-4 alpha (HNF-4α; ab41898, Abcam, Cambridge, UK), alpha-smooth muscle actin (α-SMA; ab5694, Abcam, Cambridge, UK), and Ki-67 (ab15580, Abcam, Cambridge, UK) at 4 °C for 24 h. Next, sections were incubated with biotinylated goat anti-rabbit IgG antibody (Cat. no. kit-9710; Fuzhou Maixin Biotech Co., Ltd., Fuzhou, China), followed by incubation with peroxidase-conjugated biotin-streptavidin complex (Fuzhou Maixin Biotech Co., Ltd.) for 15 min at room temperature, and lastly stained with diaminobenzidine at room temperature for 2 min and counterstained with hematoxylin at room temperature for 3–5 min. Finally, sections were photographed using an optical microscope (Olympus, Tokyo Metropolitan, Japan). The liver sections were analyzed in ten consecutive optical fields for each rat, and the positive cells were quantified using ImageJ.

### Immunofluorescence (IF) staining

When HSCs reached 60–70% confluency on 24-well plates, they were cultured with hBM-MSCs-Ex (5 ng/ml) for 48 h. Next, HSCs were incubated with 4% paraformaldehyde at room temperature for 10 min and then incubated with 1% bovine serum albumin (BSA; Biosharp, China) for 30 min. Cells were incubated with a primary antibody against α-SMA (ab5694, 1:100 dilution, Abcam, Cambridge, UK) for 1 h, followed by incubation with a secondary antibody (goat anti-rabbit IgG, ab15007, 1:500 dilution, Abcam, Cambridge, UK) for 30 min at room temperature. Rhodamine phalloidin (Thermal Scientific, USA) was stained for cytoskeleton. The nuclei were labeled with DAPI (Thermal Scientific, USA). Fluorescent images were captured using an EVOS Cell Imaging System (Thermo Scientific, USA).

### Western blotting

HSCs were co-cultured with either SFM, hBM-MSCs, or hBM-MSCs-Ex (5 ng/ml) for 48 h before samples were collected for protein extraction. Liver tissue was collected from each treatment group (liver fibrosis, hBM-MSCs, and hBM-MSCs-Ex group) for protein extraction. Protein samples were mixed with SDS sample buffer and heated to 95 °C for 10 min, followed by separation on SDS-polyacrylamide gels. Resolved proteins were electro-blotted onto nitrocellulose membrane and probed with antibodies against PPARγ (ab 23673), Wnt3a (ab 248472), Wnt10b (ab70816), β-catenin (ab32572), WISP1 (ab50041), Cyclin D1 (ab16663), α-SMA (ab5694), Collagen I (ab138492), and GAPDH (ab 8245) overnight at 4 °C (1:1000 dilution, Abcam, Cambridge, UK). Nitrocellulose membranes were then incubated with a secondary antibody, HRP-conjugated goat anti-rabbit IgG (ab15007), at room temperature for 2 h, and visualized by chemiluminescent detection according to the manufacturer’s instructions (Immobilon western chemiluminescent HRP substrate, Millipore).

### Statistical analysis

Statistical analysis was performed using GraphPad Prism Version 6. One-way ANOVA with Dunnett’s multiple comparisons test was used to test for statistically significant differences. All quantitative data are expressed as mean ± SD. *p* < 0.05 was considered to be statistically significant.

## Results

### Identification and characterization of hBM-MSCs-Ex

**Fig. 1:**
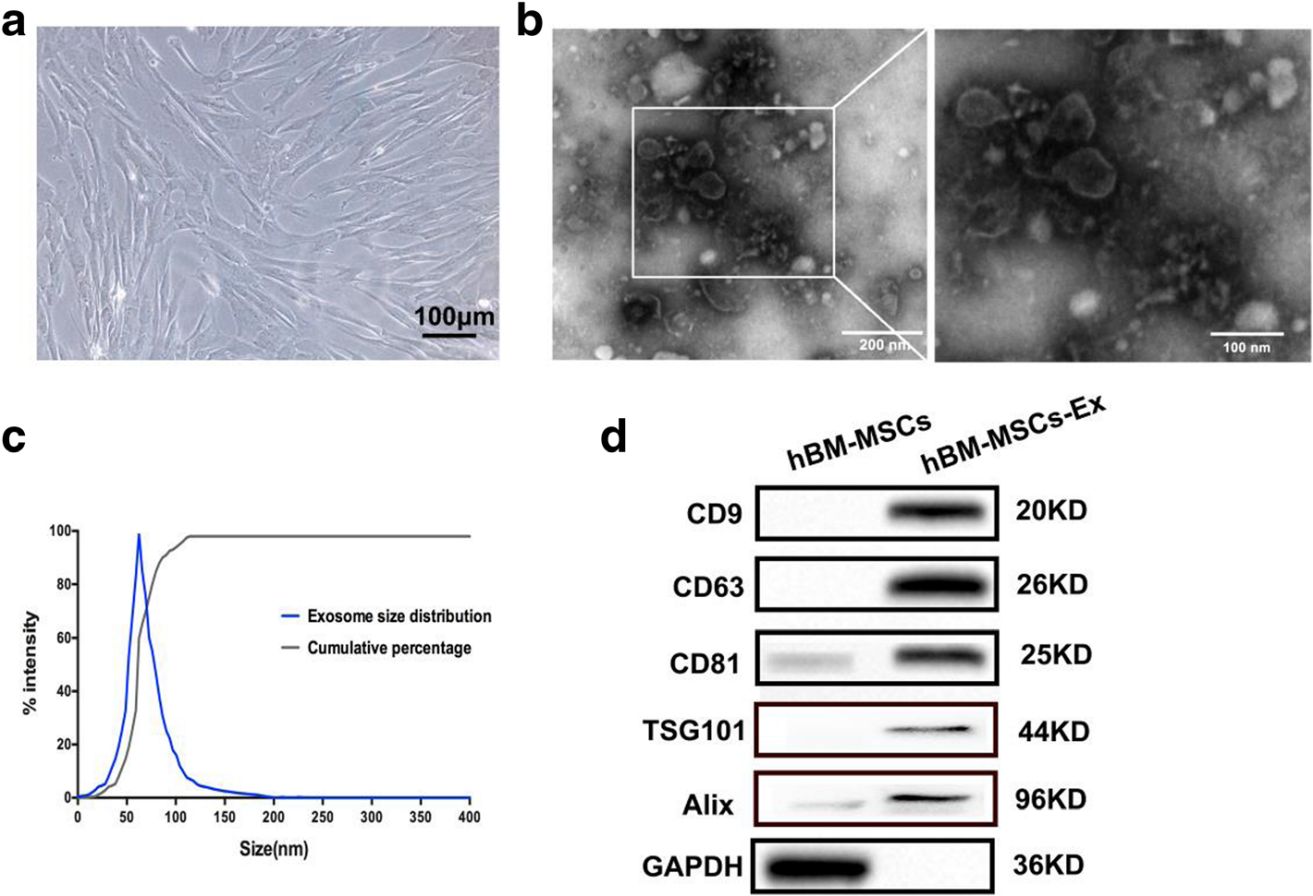
Exosome characterization. **a** Morphological appearance of cultured hBM-MSCs (bar = 100 μm). **b** Morphological analysis of hBM-MSCs-Ex by transmission electron microscopy (bar = 100 nm and 200 nm). **c** Size distribution measurements under flow conditions by nanoparticle tracking with the corresponding video frame. **d** Western blot assay indicated the positive expression of CD9, CD63, CD81, TSG101 and Alix proteins in hBM-MSCs-Ex

hBM-MSCs-Ex were successfully isolated from hBM-MSCs using the differential centrifugation method (Fig. 1a, b). We observed purified exosome morphology using transmission electron microscopy. As shown in Fig. 1b, c, the exosomes had a characteristic saucer-like shape that was limited by a lipid bilayer, with a diameter ranging from 30 to 100 nm. Western blotting confirmed that hBM-MSCs-Ex expressed, and were enriched for, the known exosomal markers CD9, CD63, CD81, TSG101 and Alix (Fig. 1d).

### hBM-MSCs-Ex alleviates CCl4-induced rats liver fibrosis

**Fig. 2:**
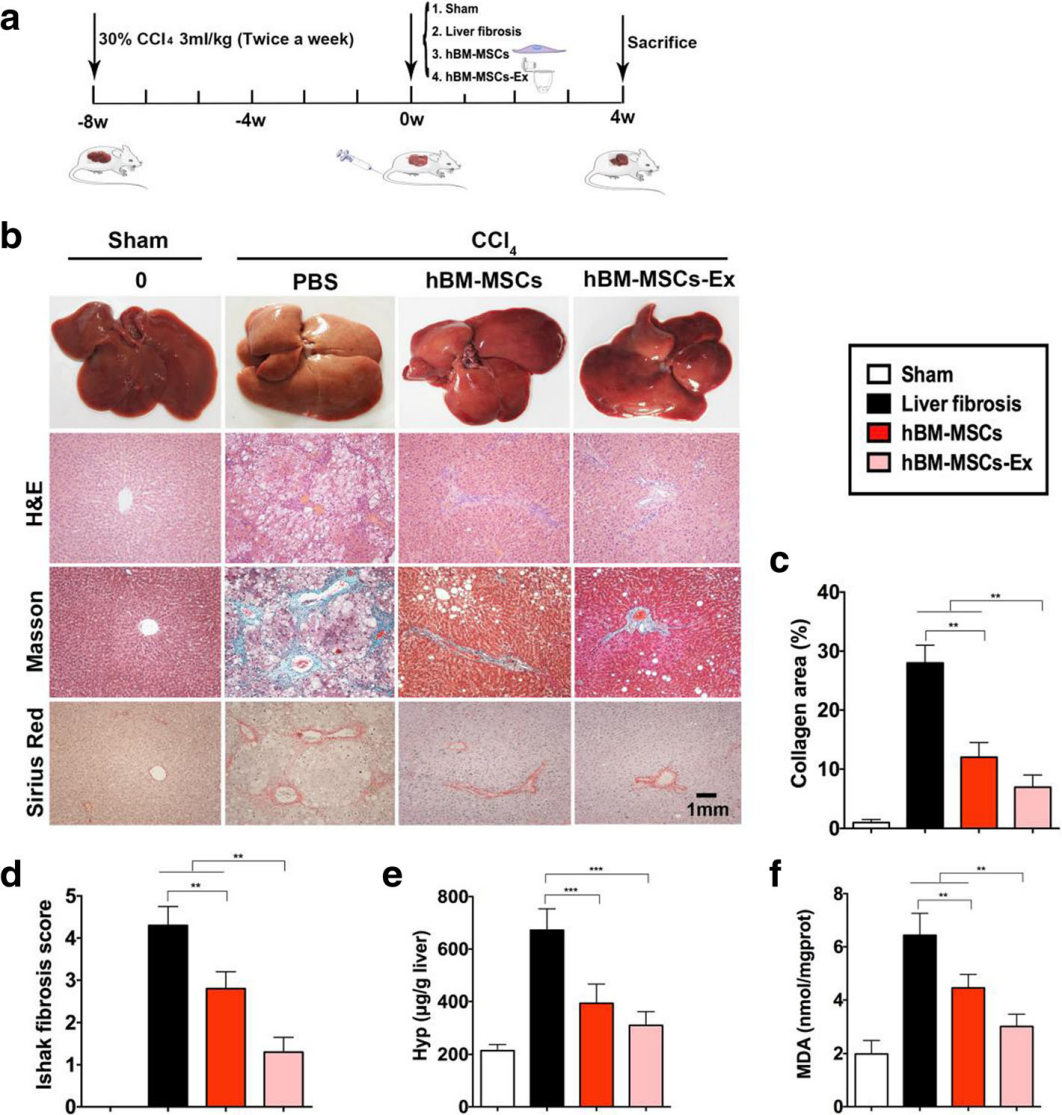
hBM-MSCs-Ex alleviates liver fibrosis in rats. **a** Study design. **b** Representative images showing the gross morphology and histological analysis of liver (scale bar = 1 mm), *n* = 12. **c** Collagen proportionate area quantification by computer-assisted image analysis at 4 weeks. **d** Ishak scoring criteria. **e**, **f** Quantitative analysis of hepatic Hyp and MDA content. **p < 0.01, ***p < 0.001. Data: *n* = 12; mean ± SD

The effectiveness of isolated hBM-MSCs-Ex to alleviate CCl_4_-induced liver fibrosis was assessed in a rat model. Visual inspection showed that the liver from sham control rats had a smooth, uniform surface and soft texture (Fig. 2). In contrast, CCl_4_ administration for 8 weeks resulted in hard, fibrous capsules covering the liver surface (Fig. 2). The liver tissue sections from CCl_4_-treated animals exhibited focal fibrosis, confirming the successful establishment of an animal model of liver fibrosis. At this point, hBM-MSCs or hBM-MSCs-Ex transplantation was performed and visual inspection of the liver was carried out after a 4-week treatment. The livers of hBM-MSCsand hBM-MSCs-Ex**-**treated rats had less fibrous capsules and were smoother and a deeper red in color than the livers of the untreated liver fibrosis group, suggesting that treatment with either hBM-MSCs or hBM-MSCs-Ex distinctly decreased fibrosis.

Histopathological examination using H&E staining, Masson’s trichrome, and Sirius red staining was performed to quantify the degree of liver fibrosis (Fig. 2b). The area of liver tissue that stained positive for collagen was significantly reduced in hBM-MSCs and hBM-MSCs-Ex-treated rats compared to the untreated liver fibrosis group. Moreover, the collagen area measured in the hBM-MSCs-Ex treatment group was lower than that measured in the hBM-MSCs treatment group (untreated liver fibrosis group, 28.2% ± 3.5%; hBM-MSCs treatment, 12.1% ± 2.4%; and hBM-MSCs-Ex treatment, 7.4% ± 1.9% respectively; Fig. 2b, c, *p* < 0.01). The Ishak fibrosis score showed a statistically significant decrease in the hBM-MSCs and hBM-MSCs-Ex-treated groups compared to the untreated liver fibrosis group. Again, the hBM-MSCs-Ex treatment group had a lower score than the hBM-MSCs treatment group (untreated liver fibrosis group, 4.3 ± 0.5; hBM-MSCs treatment, 2.8 ± 0.4; and hBM-MSCs-Ex treatment, 1.3 ± 0.4 respectively; Fig. 2d, *p* < 0.01).

Next, we analyzed the levels of hydroxyproline (Hyp) and malondialdehyde (MDA) which indicate the presence of liver collagen fibers and lipid peroxidation changes. Hyp is a non-essential amino acid and a main component in collagen tissue. MDA is an end product of membrane lipid peroxidation, and its level can be used as a marker for oxidative stress and liver cell injury [4]. Compared to the untreated liver fibrosis group, the Hyp and MDA levels were significantly reduced in hBM-MSCs and hBM-MSCs-Ex-treated rats. In addition, the Hyp and MDA levels in the hBM-MSCs-Ex treatment group were lower than those in the hBM-MSCs treatment group (For Hyp: untreated liver fibrosis group, 671.2 ± 78.4; hBM-MSCs treatment, 392.5 ± 45.6; and hBM-MSCs-Ex treatment, 364.8 ± 30.6μg/g respectively. For MDA: untreated liver fibrosis group, 6.4 ± 0.7; hBM-MSCs treatment, 4.3 ± 0.4; and hBM-MSCs-Ex treatment, 3.1 ± 0.3 nmol/mgport respectively; Fig. 2e, f, *p* < 0.01, *p* < 0.001). These results suggest that hBM-MSCs-Ex-treatment significantly alleviates liver fibrosis in CCl_4_-induced rats and that the therapeutic effect of hBM-MSCs-Ex-treatment was greater than that of hBM-MSCs treatment for some indicators.

### hBM-MSCs-Ex treatment reduces liver inflammation and improves liver function

**Fig. 3:**
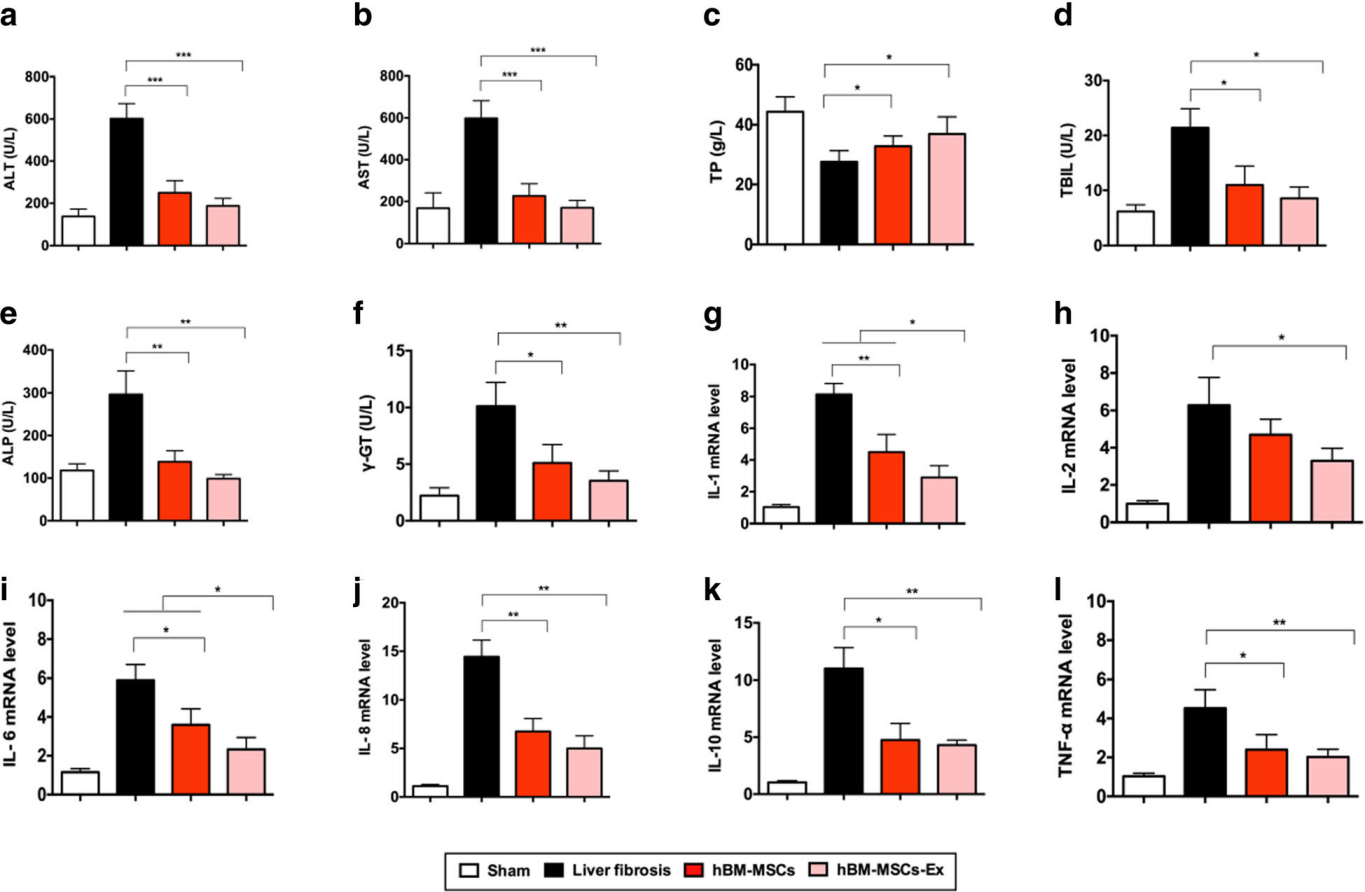
hBM-MSCs-Ex improves liver function and decreases inflammation. **a** ALT, alanine aminotransferase. **b** AST, aspartate aminotransferase. **c** TP, total protein. **d** TBIL, total bilirubin. **e** ALP, alkaline phosphatase. **f** γ-GT, gamma glutamyl transpeptidase. **g–l** The relative inflammatory gene expression for IL-1, IL-2, IL-6, IL-8, IL-10, and TNF-α. *p < 0.05, **p < 0.01, ***p < 0.001. Data: *n* = 12; mean ± SD

Biochemical analyses were performed to assess the restoration of liver function after hBM-MSCs and hBM-MSCs-Ex treatment. The serum levels of ALT, AST, TBIL, ALP, and γ-GT were significantly suppressed in the hBM-MSCs and hBM-MSCs-Ex-treated groups (Fig. 3, *p* < 0.05). In addition, the serum level of TP in hBM-MSCs and hBM-MSCs-Ex-treated groups was higher than that in the untreated liver fibrosis group (Fig. 3c, *p* < 0.05). These results suggest enhanced liver function in the two hBM-MSCs and hBM-MSCs-Ex treatment groups.

Next, we detected the expression of inflammatory factors in liver tissue by qRT-PCR. Compared to the untreated liver fibrosis group, the expression of inflammatory factors including IL-1, IL-2, IL-6, IL-8, IL-10, and TNF-α were significantly decreased in the hBM-MSCs and hBM-MSCs-Ex treatment groups (Fig. 3g–l, *p* < 0.05). Interestingly, we found that the expression of IL-1and IL-6 in hBM-MSCs-Ex treatment group was significantly decreased when compared to the hBM-MSCs treatment group (Fig. 3g, i; *p* < 0.05). Therefore, both hBM-MSCs and hBM-MSCs-Ex treatment had an anti-fibrosis effect, as evidenced by decreased liver collagen, together with improvement of liver function and reduction of inflammation.

### hBM-MSCs-Ex treatment promote hepatocyte regeneration and inhibits α-SMA expression

**Fig. 4:**
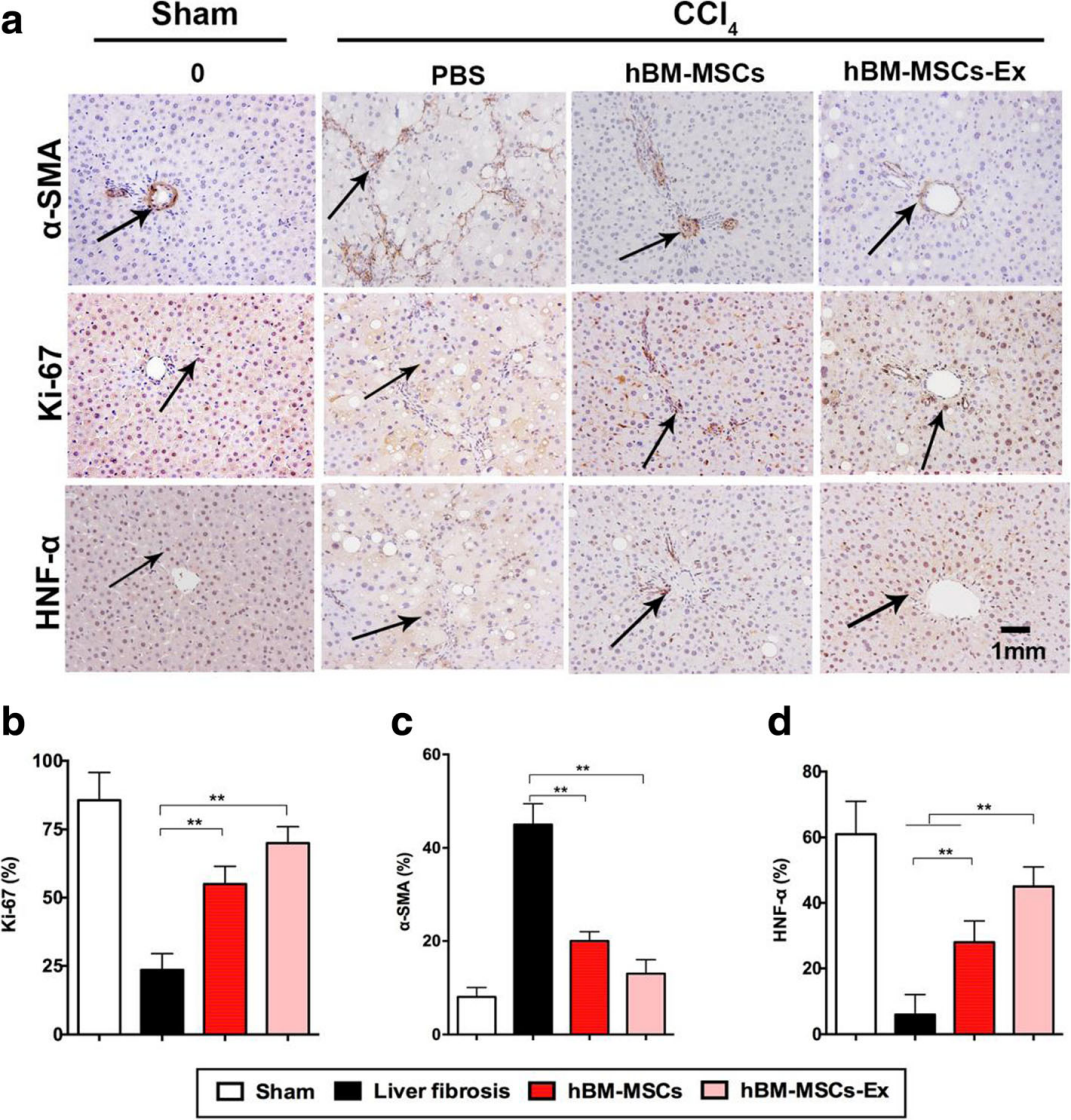
hBM-MSCs-Ex increases Ki-67^+^ and decreases α-SMA^+^ in a rat model of liver fibrosis. **a** Photomicrographs of liver tissue sections showing IHC staining for α-SMA and Ki-67 (black arrows indicate brown-positive cells, bar = 1 mm). **b**–**d** Quantification of α-SMA and Ki-67-positive cells was performed by computer-assisted image analysis. **p < 0.01, ***p < 0.001. Data: *n* = 12; mean ± SD

To evaluate whether hBM-MSCs-Ex treatment could enhance hepatocyte proliferation in cirrhotic liver, Ki-67 expression levels were assessed by IHC (Fig. 4a, b). In treated rat liver, the percentage of Ki-67^+^ cells in hBM-MSCs (55.14%) and hBM-MSCs-Ex (71.28%) treatment groups was increased significantly when compared to the untreated liver fibrosis group (23.29%, *p* < 0.01, *p* < 0.001). HNF-4α was a key nuclear receptor protein required for liver development [27]. The percentage of HNF-4α^+^ cells in hBM-MSCs-Ex (45.18%) group was increased significantly when compared with the liver fibrosis (28.54%) and hBM-MSCs group (6.46%, Fig. 4a, d; *p* < 0.01). To further investigate the mechanism underlying the hBM-MSCs and hBM-MSCs-Ex-mediated rescue of CCl_4_-damaged liver, we examined the expression level of α-SMA, which is a key cytokine involved in the development of liver fibrosis and HSC activation (Fig. 4a, c). The expression of α-SMA^+^ was significantly decreased in the hBM-MSCs (21.65%) and hBM-MSCs-Ex (13.84%) treatment groups when compared with the untreated liver fibrosis group (45.37%, *p* < 0.01). The above results indicate that hBM-MSCs-Ex promote anti-fibrosis by stimulating hepatocyte regeneration and inhibiting α-SMA expression on liver fibrosis.

### hBM-MSCs-Ex treatment inhibits HSC activation through inhibition of the Wnt/β-catenin signaling pathway both in vivo and in vitro

**Fig. 5:**
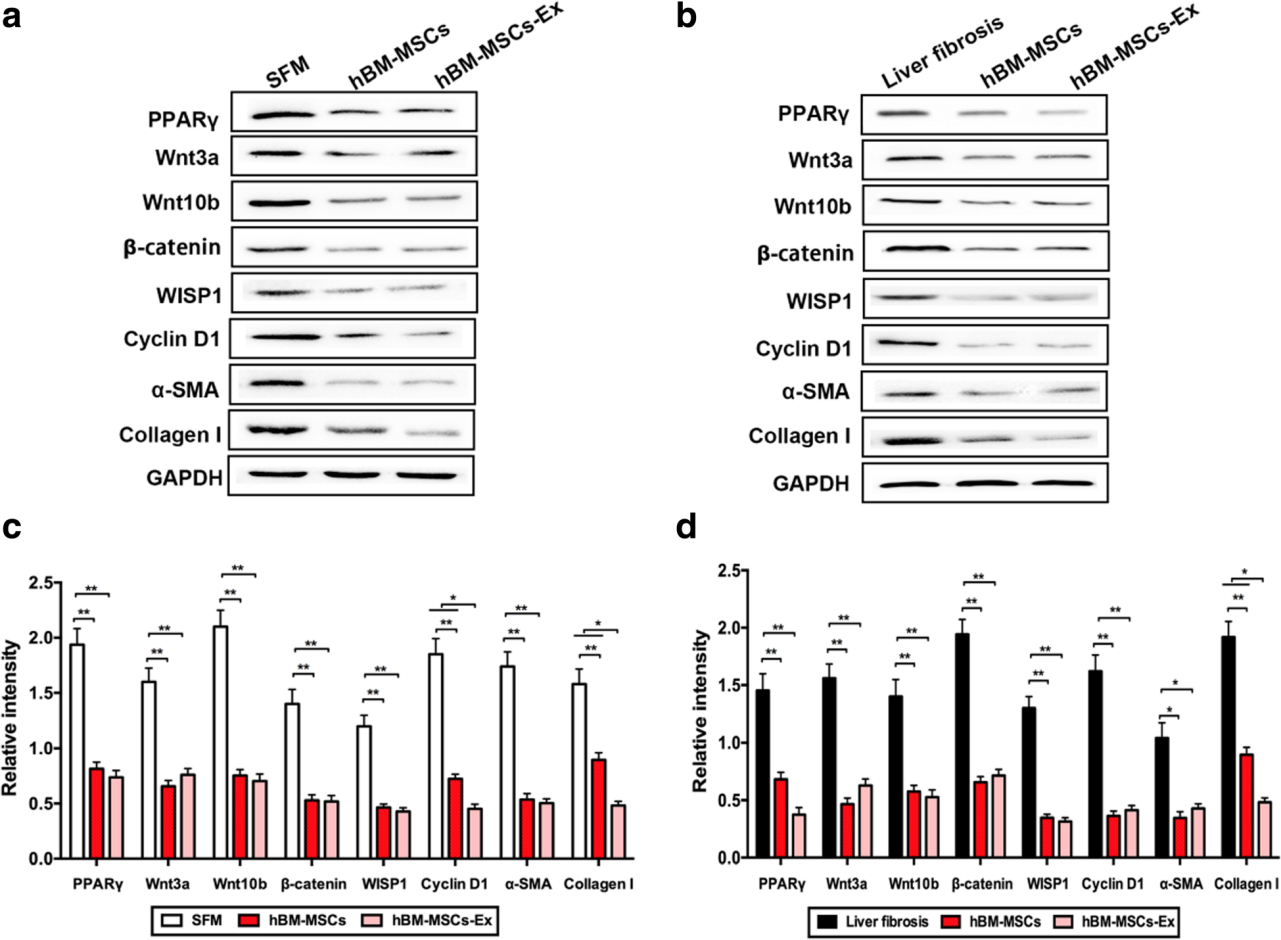
hBM-MSCs-Ex inhibits the Wnt/β-catenin signaling pathway. **a**, **c** Representative western blotting analysis measuring PPARγ, Wnt3a, Wnt10b, β-catenin, WISP1, Cyclin D1, α-SMA, and Collagen I in HSCs and liver fibrosis tissue. **b**, **d** The quantification of protein relative intensity. ***p < 0.001, ***p < 0.001. Data, *n* = 3; mean ± SD

To further explore the association between the anti-fibrosis effect of hBM-MSCs-Ex treatment and the Wnt signaling pathway in liver fibrosis, we examined the expression levels of proteins involved in the Wnt/β-catenin signaling pathway, both in HSCs and in liver fibrosis tissues, through western blotting (Fig. 5a, c). Both hBM-MSCs and hBM-MSCs-Ex treatment significantly inhibited the expression of PPARγ, Wnt3a, Wnt10b, β-catenin, WISP1, Cyclin D1, α-SMA, and Collagen I in both HSCs and liver fibrosis tissues (Fig. 5b, d; *p* < 0.005, *p* < 0.001). Interestingly, the expression of Collagen I on hBM-MSCs-Ex treatment was significantly decreased compared to hBM-MSCs treatment, in both HSCs and liver fibrosis tissues (Fig. 5b, d; *p* < 0.005). In addition, the expression of Cyclin D1 on hBM-MSCs-Extreatment was significantly decreased compared to that in HSCs (Fig. 5b, *p* < 0.005).

## Discussion

In this study, we have demonstrated that hBM-MSCs-Ex have the ability to alleviate liver fibrosis, including the recovery of markers associated with improved liver function, inhibition of inflammation, and increased hepatocyte regeneration. Moreover, the therapeutic effect of hBM-MSCs-Ex appears enhanced over the effect of the parent hBM-MSCs. Furthermore, we found that, both in vitro and in vivo, the hBM-MSCs-Ex inhibit HSC activation via inhibition of Wnt/β-catenin signaling.

MSCs-derived exosomes have been reported to have therapeutic properties on hepatic disease [27]. Recently, studies have demonstrated that MSCs-derived exosomes are as effective as their parent stem cells in promoting liver repair and regeneration [28, 29]. Exosomes are believed to enhance liver function and/or alleviate pathological phenotypes through the transfer of their cargo to injured cells [30, 31]. In this study, we successfully isolated exosomes from hBM-MSCs and characterized the hBM-MSCs-Ex. Our CCl_4_-induced liver fibrosis model demonstrated that administration of hBM-MSCs-Ex effectively alleviated liver fibrosis. Furthermore, we found that hBM-MSCs-Ex could aid in the recovery of liver functionality and increase hepatocyte regeneration. This is consistent with reports previously showing that human umbilical cord MSC-derived exosomes (hucMSC-Ex) could ameliorate CCl_4_-induced liver fibrosis and provide a protective effect on hepatocytes [32]. In addition, MSCs-derived exosomes can inhibit inflammation and contribute to hepatocyte regeneration [33]. Our studies found that the hBM-MSCs-Ex significantly decreased inflammatory cytokines, which are one of the main factors inducing liver fibrosis. We speculate that hBM-MSCs-Ex promote liver tissue repair by reducing the inflammatory response.

Although both hBM-MSCs and hBM-MSCs-Ex groups can reduce liver fibrosis in the study, exosomes have several special characteristic compared to their parent cells. The reasons that exosomes may offer advantages over their parental cells may be as follows: (1) they are smaller in size and less complex than their parent cells and, thus, easier to produce and store [8]; (2) they are devoid of viable cells and, as such, present no risk for tumor formation [8]; (3) they are less immunogenic than their parent cells due to the lower content of membrane-bound proteins [9]; and (4) they are safe cell-free reagents with controllable outcomes [9]. Based on the above advantages, we speculate that hBM-MSCs-Ex may serve as a new therapeutic strategy for liver fibrosis. In our study, treatment with hBM-MSCs-Ex resulted in significantly lower fibrosis indicators than hBM-MSCs, including collagen area, Ishak fibrosis score, MDA, IL-1, and IL-6 (Figs. 2 and 3, *p* < 0.05). These results indicate that the therapeutic effect of hBM-MSCs-Ex against liver fibrosis was greater than that of hBM-MSCs.

**Fig. 6:**
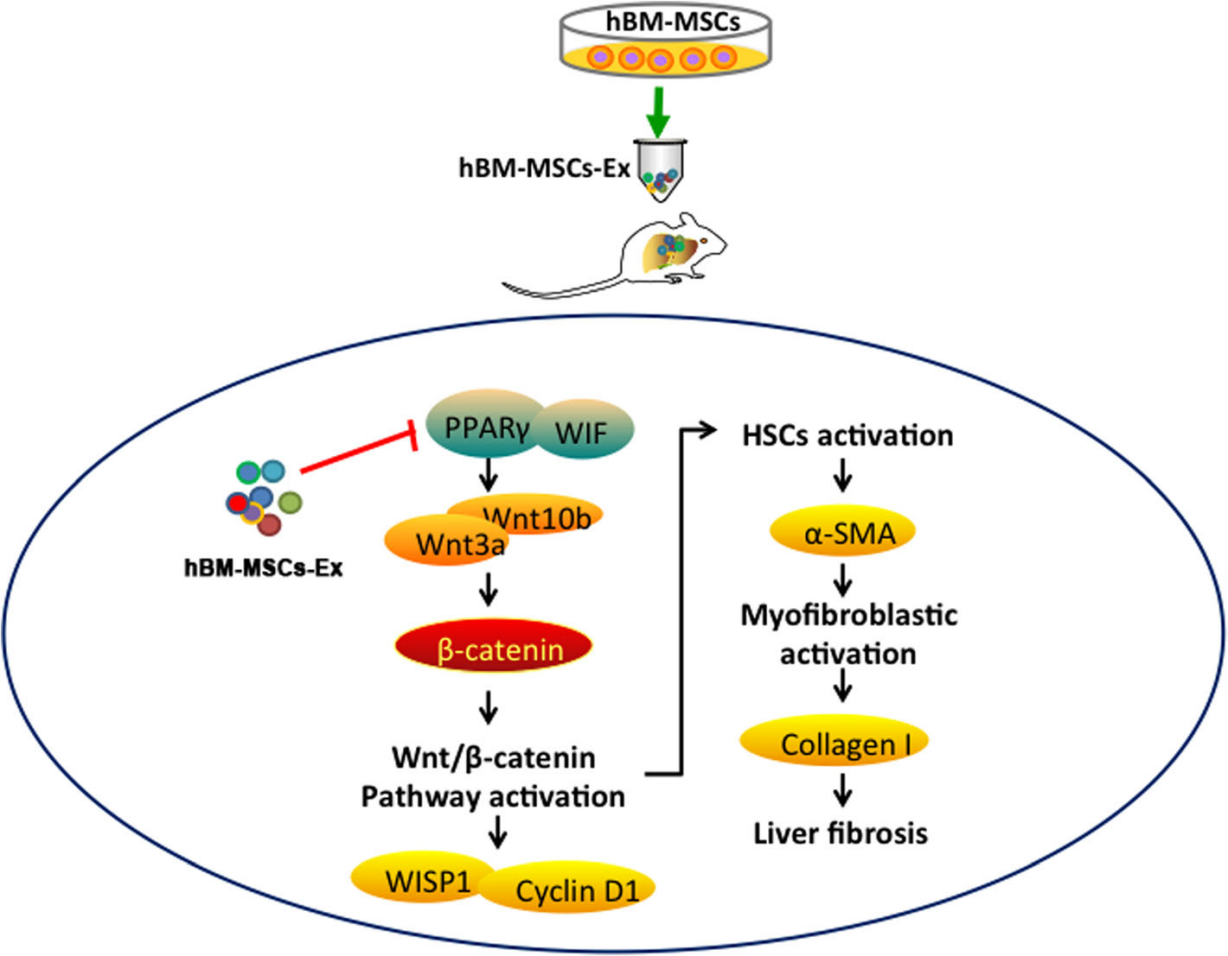
hBM-MSCs-Ex treatment alleviates liver fibrosis in both HSCs and liver fibrosis tissues through inhibition of Wnt/β-catenin signaling (PPARγ, Wnt3a, Wnt10b, β-catenin) and the downregulation of downstream gene expression (WISP1, Cyclin D1). This inhibits HSC activation to prevent further liver fibrosis

It has been reported that the inhibition of Wnt/β-catenin signaling results in the downregulation of HSC activation and an eventual reduction in CCl_4_-induced liver fibrosis [34]. In our study, the results showed that hBM-MSCs-Ex treatment can downregulate the expression of several proteins (PPARγ, Wnt3a, Wnt10b, β-catenin) in the Wnt signaling pathway, and this subsequently contributes to inhibition of downstream gene expression (WISP1, Cyclin D1). As a consequence, HSC and myofibroblastic activation was inhibited, leading to reduce liver fibrosis (Figs. 5 and 6). A previous study suggests the activated Wnt/β-catenin signaling pathway can promote the transcription of certain genes, specifically, WISP1 and Cyclin D1, which have been shown to play an important role in HSC proliferation, metastasis, and ECM formation [35]. In addition, the expression of α-SMA in liver tissue was used as an indicator of HSCs activation, and its upregulation is implicated in the occurrence and development of hepatic fibrosis [36, 37]. We found that the hBM-MSCs-Ex treatment significantly decreased of α-SMA expression level both in vivo and in vitro (Figs. 4 and 5). Inhibition of HSC proliferation and activation and subsequent prevention of the fibrotic myofibroblast phenotype, results in collagen degradation and reduced liver fibrosis. In the present study, the levels of Collagen I decreased in hBM-MSCs-Ex treatment group (Fig. 5). These data demonstrate that hBM-MSCs-Ex treatment can alleviate liver fibrosis through inhibition of the Wnt/β-catenin signaling pathway.

## Conclusion

Altogether, our results clearly demonstrate that hBM-MSCs-Ex treatment alleviates liver fibrosis in vivo. Moreover, administration of hBM-MSCs-Ex reduces liver fibrosis via inhibition of Wnt/β-catenin signaling to prevent HSC activation. Therefore, the use of hBM-MSCs-Ex presents a new and promising therapeutic strategy for hepatic disease in the clinical setting.

## Additional files

Additional file 1: **Figure S1.** The rat body weight change in CCl_4_-induced liver fibrosis, *n* = 12. (DOCX 175 kb)

Additional file 2: **Figure S2.** The rat organ index of liver, kidney and spleen in CCl_4_ -induced liver fibrosis, **p < 0.01, *n* = 12. (DOCX 114 kb)

## Abbreviations

ALP: Alkaline phosphatase
ALT: Alanine aminotransferase
AST: Aspartate aminotransferase
ECM: Extracellular matrix
hBM-MSCs: Human bone marrow-derived mesenchymal stem cells
hBM-MSCs-Ex: Human bone marrow mesenchymal stem cells-derived exosomes
HNF-4α: Hepatocyte nuclear factor-4 alpha
HSCs: Hepatic stellate cells
Hyp: Hydroxyproline
IF: Immunofluorescence
IHC: Immunohistochemistry
MDA: Malonaldehyde
MSCs: Mesenchymal stem cells
qRT-PCR: Quantitative real-time PCR
TBIL: Total bilirubin
TP: Total protein
α-SMA: Alpha-smooth muscle actin
γ-GT: Gamma glutamyl transpeptidase
