# Supplementary figures and images for: Human bone marrow mesenchymal stem cells-derived exosomes alleviate liver fibrosis through the Wnt/β-catenin pathway
Source: Stem Cell Res Ther. 2019 Mar 18;10:98. doi: 10.1186/s13287-019-1204-2 (PMC6421647; doi:10.1186/s13287-019-1204-2)

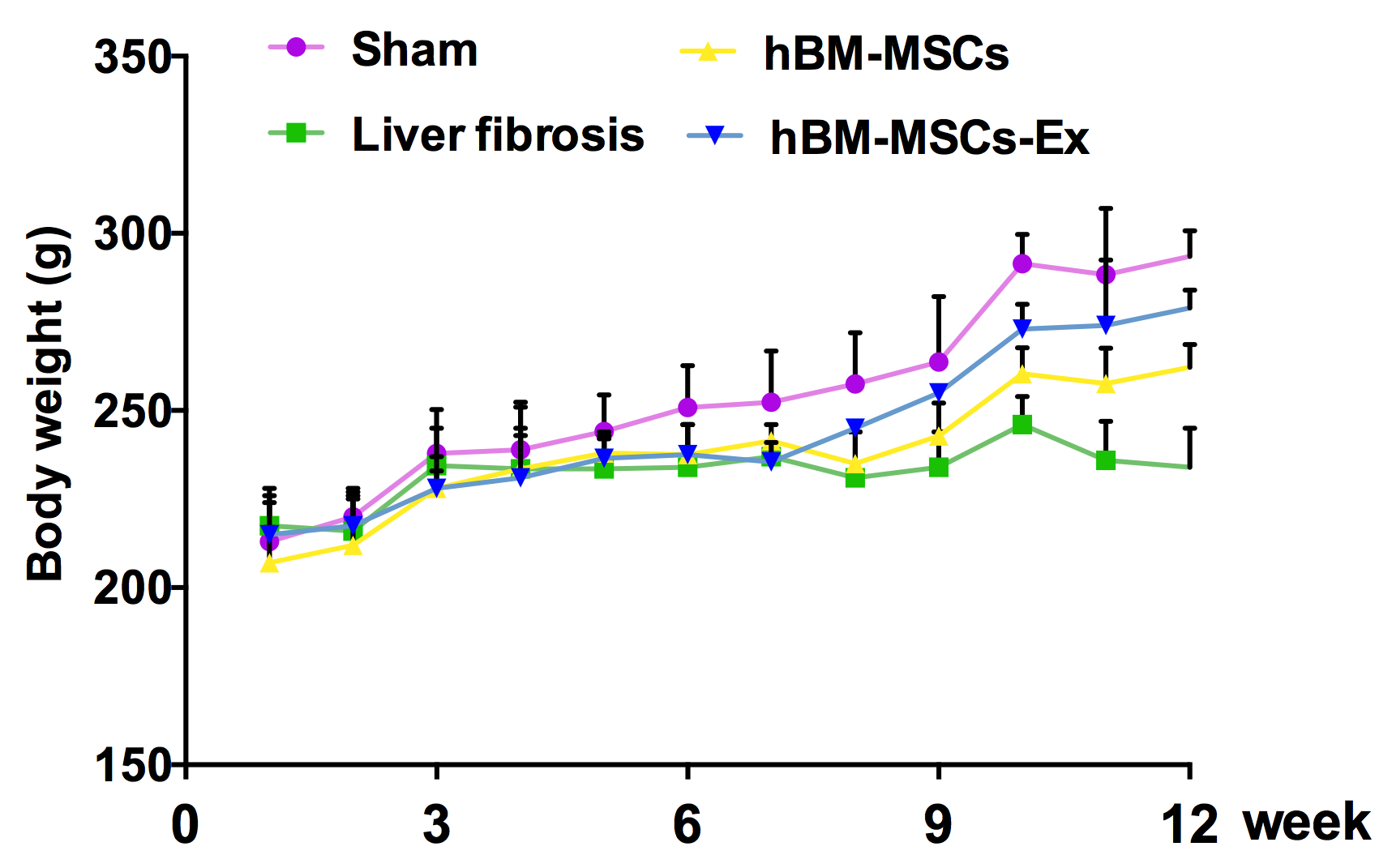


**Additional file 1: Figure S1.** The rat body weight change in CCl4-induced liver fibrosis, n=12.

Supplement: Supplementary file 1 — Figure S1. The rat body weight change in CCl4-induced liver fibrosis, n = 12. (DOCX 175 kb) [file 13287_2019_1204_MOESM1_ESM.docx]
