# Supplementary material for: Human bone marrow mesenchymal stem cells-derived exosomes alleviate liver fibrosis through the Wnt/β-catenin pathway
Source: Stem Cell Res Ther. 2019 Mar 18;10:98. doi: 10.1186/s13287-019-1204-2 (PMC6421647; doi:10.1186/s13287-019-1204-2)
Supplement: Supplementary file 2 — Figure S2. The rat organ index of liver, kidney and spleen in CCl4 -induced liver fibrosis, **p < 0.01, n = 12. (DOCX 114 kb) [file 13287_2019_1204_MOESM2_ESM.docx]

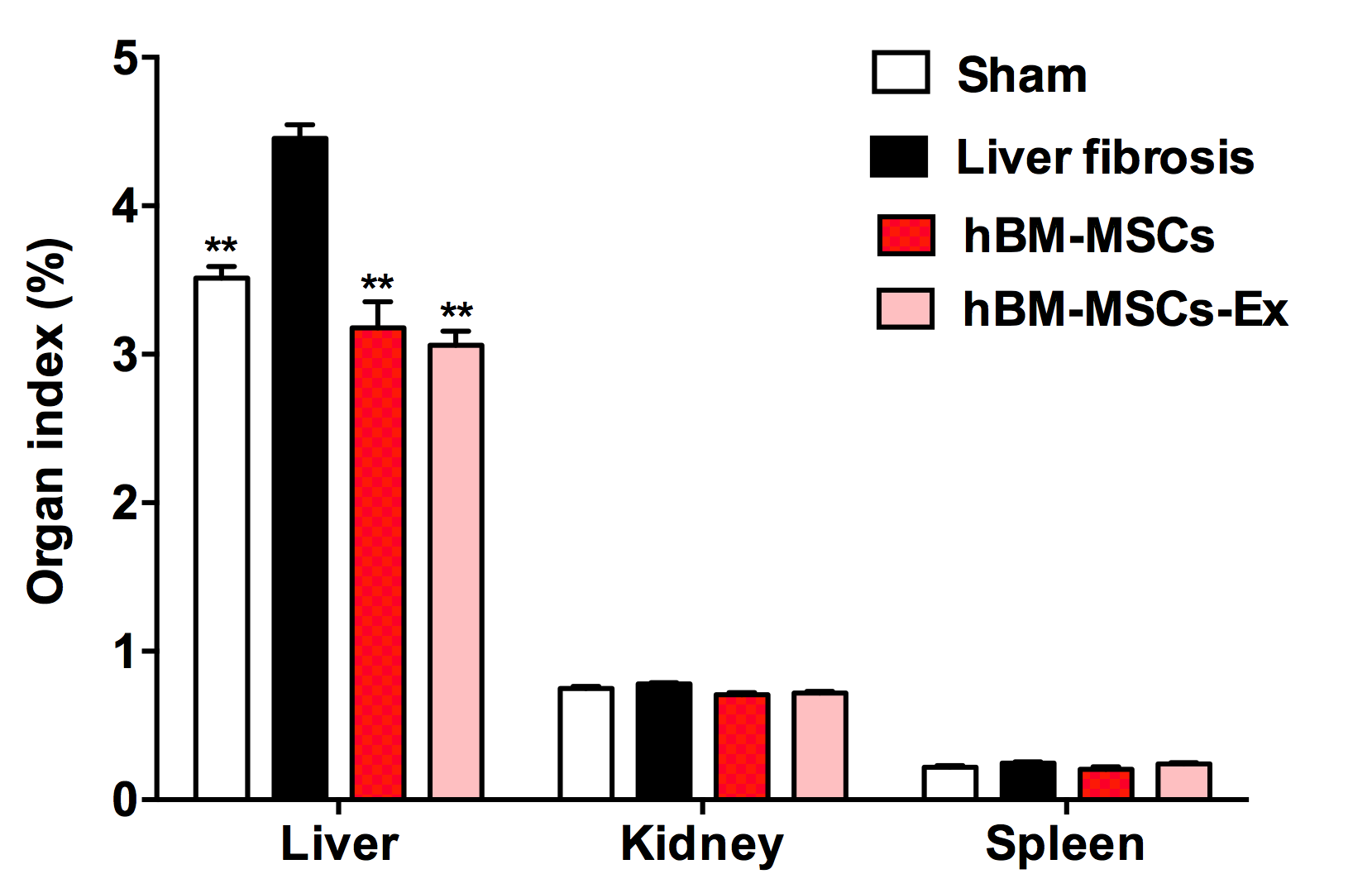


**[Additional file 2:](https://static-content.springer.com/esm/art%3A10.1186%2Fs13287-018-1045-4/MediaObjects/13287_2018_1045_MOESM1_ESM.tif) Figure S2.** The rat organ index of liver kidney and spleen in CCl4-induced liver fibrosis, ***p*<0.01 when compared to the liver fibrosis group, n=12.
